# Maternal Cardiovascular Disease After Pre-Eclampsia and Gestational Hypertension: A Narrative Review

**DOI:** 10.1177/15598276211037964

**Published:** 2021-12-27

**Authors:** Clare Oliver-Williams, Jasmine D. Johnson, Catherine J. Vladutiu

**Affiliations:** 61444 From Homerton College, University of Cambridge, Cambridge, UK; Cardiovascular Epidemiology Unit, Department of Public Health and Primary Care, 12204 University of Cambridge, Cambridge, UK; Department of Health Sciences, 4488 University of Leicester, Leicester, UK; Department of Obstetrics and Gynecology, 6797 University of North Carolina, Chapel Hill, North Carolina

**Keywords:** cardiovascular disease, gestational hypertension, pre-eclampsia, pregnancy, coronary heart disease, stroke, heart failure

## Abstract

Previous literature has highlighted that women who have a pregnancy affected by gestational hypertension or preeclampsia are at higher risk of cardiovascular disease (CVD) in later life. However, CVD is a composite of multiple outcomes, including coronary heart disease, heart failure, and stroke, and the risk of both CVD and hypertensive disorders of pregnancy varies by the population studied. We conducted a narrative review of the risk of cardiovascular outcomes for women with prior gestational hypertension and pre-eclampsia. Previous literature is summarized by country and ethnicity, with a higher risk of CVD and coronary heart disease observed after gestational hypertension and a higher risk of CVD, coronary heart disease and heart failure observed after pre-eclampsia in most of the populations studied. Only one study was identified in a low- or middle-income country, and the majority of studies were conducted in white or mixed ethnicity populations. We discuss potential interventions to mitigate cardiovascular risk for these women in different settings and highlight the need for a greater understanding of the epidemiology of CVD risk after gestational hypertension and pre-eclampsia outside of high-income, white populations.

…women with gestational hypertension or pre-eclampsia should be aware of their higher CVD risk…

## Introduction

Hypertensive disorders of pregnancy (HDP) occur in 8–13% of pregnancies.^1,2^ There are four distinct HDP: gestational hypertension (GH); pre-eclampsia (PE), which can progress to eclampsia; chronic hypertension; and PE superimposed on chronic hypertension. This review focuses on PE and GH, as defined in Table 1.

**Table 1.** Diagnostic Classification of the Four Hypertensive Disorders in Pregnancy.

Hypertensive disorder of pregnancy	Diagnostic criteria
Pre-eclampsia/eclampsia	Pre-eclampsia-De novo hypertension after gestational week 20, and new onset of one or more of the following: Proteinuria (protein in the urine), renal insufficiency, liver disease, neurological problems, and hematological disturbances or fetal growth restriction
	Eclampsia is a convulsive condition associated with pre-eclampsia
Gestational hypertension	New hypertension (≥ 140 mmHg systolic or ≥ 90 mmHg diastolic) presenting after 20 weeks of pregnancy without proteinuria
Chronic hypertension*	Hypertension (≥ 140 mmHg systolic or ≥ 90 mmHg diastolic) present at the booking visit, or before 20 weeks, or antihypertensive medication prescription when referred to maternity services
Pre-eclampsia superimposed on chronic hypertension*	Hypertension present before 20 weeks of gestation or pre-pregnancy, as indicated by blood pressure measurements or antihypertensive medication prescription, with one or more of the following conditions after 20 weeks gestation: Proteinuria, renal insufficiency, liver disease, neurological problems, hematological disturbances, or fetal growth restriction

*Not the focus of this review.

Definitions *from: Hypertension in pregnancy: diagnosis and management. NICE guideline* [*NG133]*^63^ *and the International Society for the Study of Hypertension in Pregnancy*^64^

PE and GH are leading causes of maternal and infant morbidity and mortality worldwide.^3^ Their prevalence varies within and between countries. The reported prevalence of PE and GH in different continents is shown in Table 2, with the highest prevalence of both PE and GH found in Africa.

**Table 2.** The Range of Reported Prevalence Estimates for Gestational Hypertension and Pre-Eclampsia in Prior Studies.

*Continent*	Pre-eclampsia	Gestational hypertension
*Africa*	0.5–10.5	0.3–28.9
*Asia*	0.2–6.7	1.8–3.4
*Australasia*	2.6–9.2	5.7–8.2
*Europe*	1.6–5.2	0.9–5.8
*North America*	1.5–4.0	3.0–8.0
*South America and the Caribbean*	1.8–7.7	None found

PE and GH are also associated with a higher risk of maternal cardiovascular disease (CVD) in later life.^4^ CVD is a group of diseases affecting the heart or blood vessels. Many are related to atherosclerosis, which is the build-up of plaque in artery walls, causing narrowing and restricted blood flow. Common forms of CVD include coronary heart disease (CHD), heart failure, and stroke. Collectively, CVD is the leading cause of mortality for women worldwide.^3^ However, CVD prevalence varies widely between countries, with individuals in low- and middle-income countries (LMIC) more likely to be affected than those in high-income countries (HIC). The highest age-standardized CVD prevalence for men and women combined is in Oman and Mozambique with over 11,501 cases per 100,000 persons. In contrast, Canada, the United States, most Western European countries, South Korea, Japan, Australia, New Zealand, Chile, Argentina, Peru, Ecuador, and Uruguay have fewer than 5600 cases per 100,000 persons.^5^ A consequence of this is that the impact of CVD is also greatest in LMIC.

**Figure 1.**
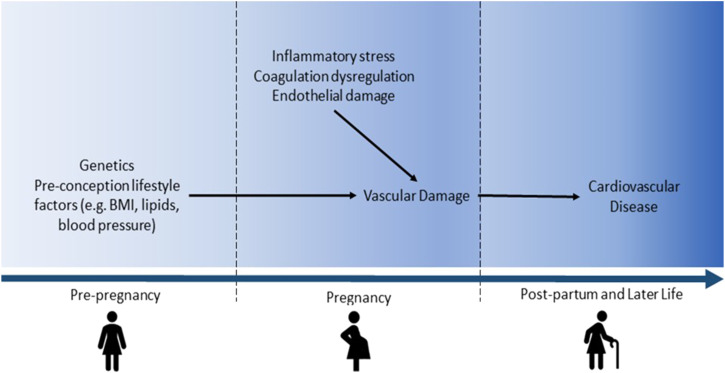
Pathophysiological mechanisms linking the development of cardiovascular disease in later life to gestational hypertension or pre-eclampsia in a prior pregnancy.

It has been suggested that there are overlapping pathophysiological mechanisms underlying GH, PE, and CVD, such as immune system activation and inflammation^6^ (Figure 1). The prevalence of these shared risk factors for CVD and GH or PE, including high BMI and chronic conditions such as polycystic ovary syndrome and kidney injury (Supplementary Figure 1), also varies between countries.

This narrative review summarizes the evidence for the higher risk of maternal CVD after GH or PE. Relevant studies are described by country and ethnicity, with a focus on overall CVD and common outcomes, including CHD, heart failures, and stroke. We discuss underlying mechanisms, and potential interventions to mitigate CVD risk among different settings and highlight future areas of research.

## Methods

A literature search was conducted in PubMed and Web of Science, using the keywords “cardiovascular disease,” “coronary heart disease,” “stroke,” “heart failure,” “gestational hypertension,” “hypertensive disorders of pregnancy,” and “pre-eclampsia.” The search was limited to studies published in English before May 2020.

Studies were considered eligible for inclusion if they assessed risk of CVD, CHD, stroke, or heart failure after either gestational hypertension or pre-eclampsia in women. Gestational hypertension and pre-eclampsia could not be combined as a single exposure.

Information was extracted from eligible studies, including author, year, and country of study; number of participants; ethnicity of participants; exposure, outcome, and relative risk (RR); and 95% confidence intervals (CI). Results are reported narratively by study location.

### Cardiovascular Diseases After Gestational Hypertension or Pre-Eclampsia

**Figure 2.**
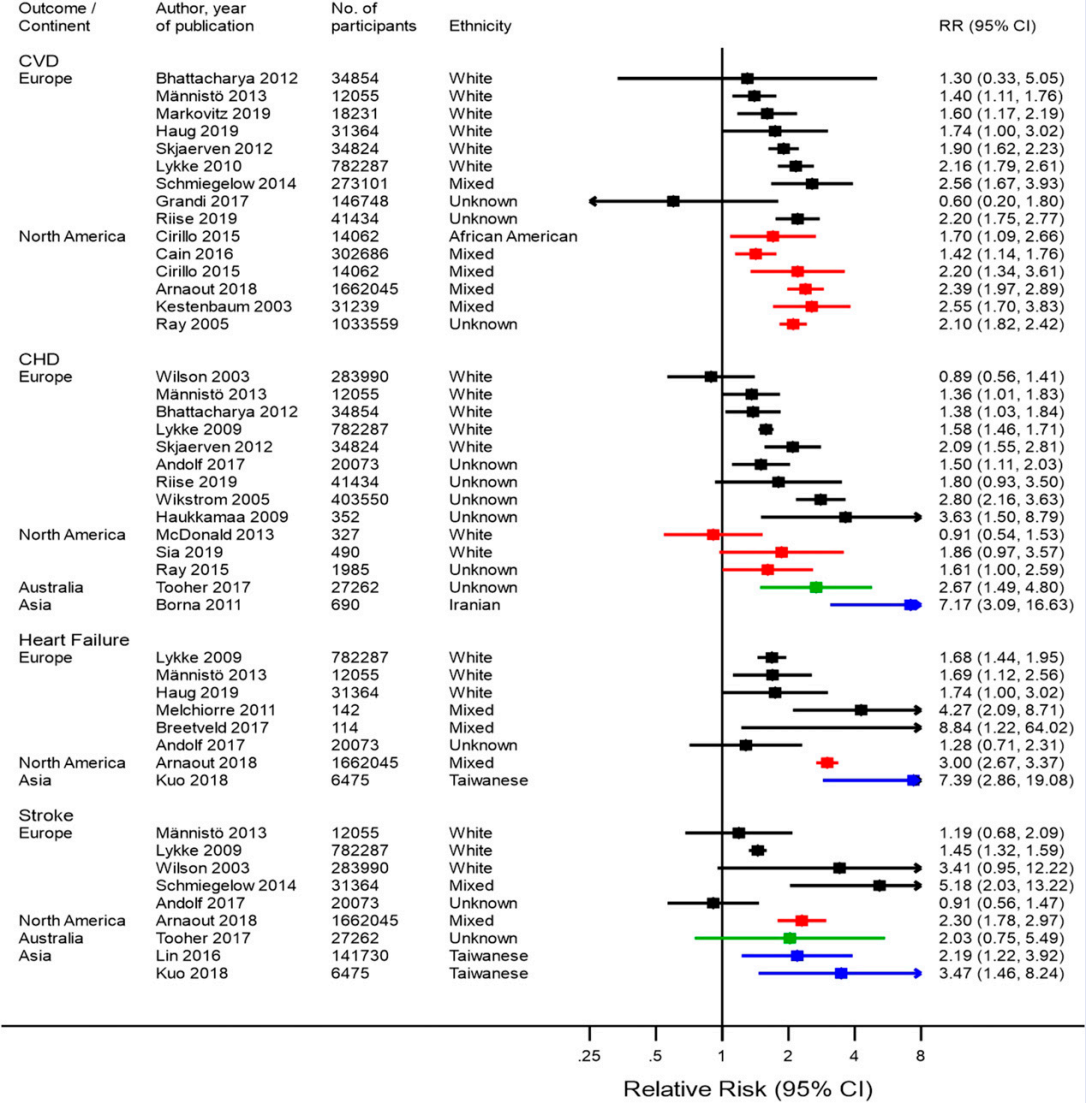
Results of studies assessing risk of cardiovascular disease, coronary heart disease, heart failure, and stroke after pre-eclampsia, by continent. Black–European studies, red–North American studies, green–Australian studies, and blue–Asian studies.

**Figure 3.**
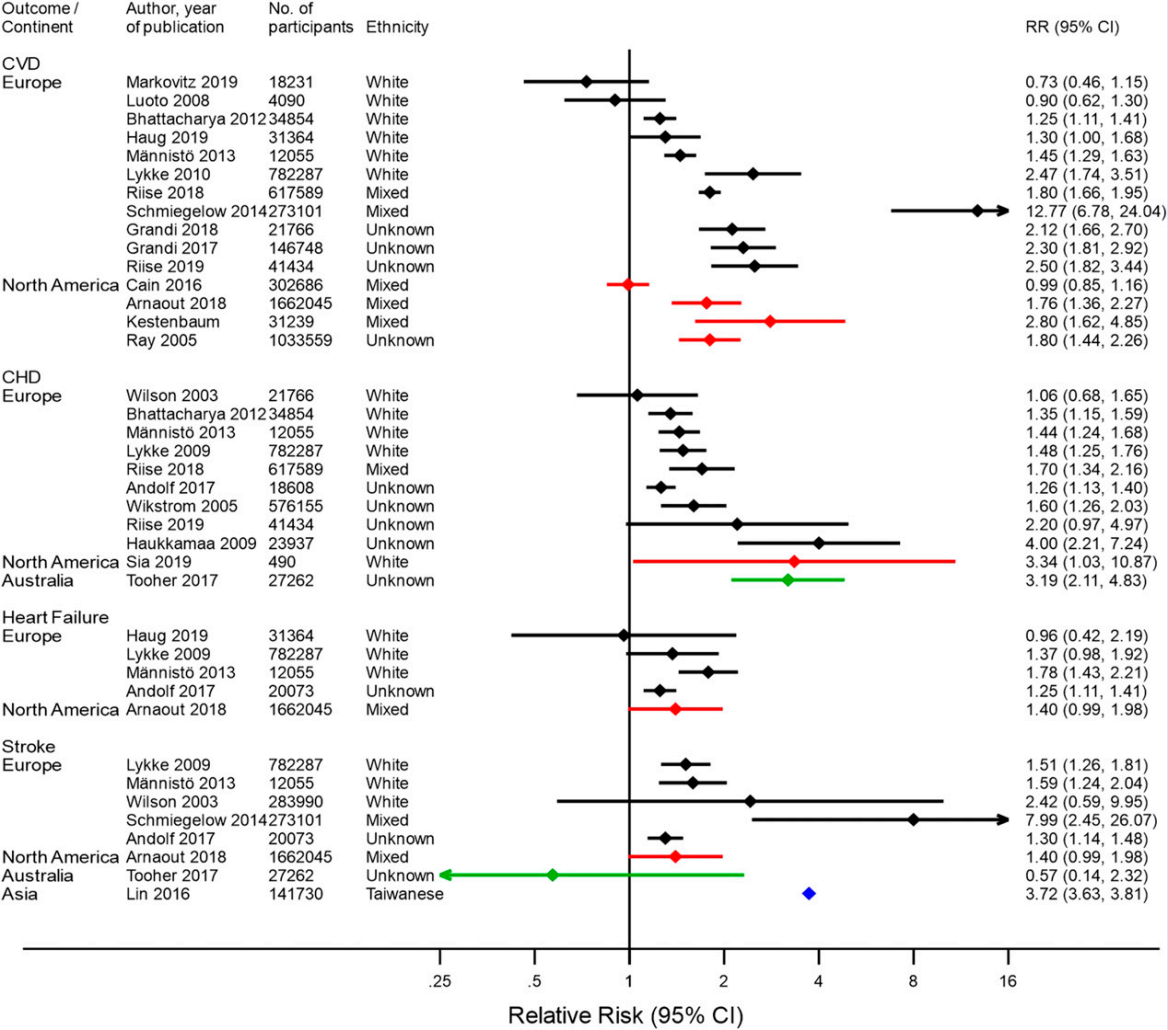
Results of studies assessing risk of cardiovascular disease, coronary heart disease, heart failure, and stroke after gestational hypertension, by continent. Black-European studies, Red-North American studies, green-Australian studies, blue-Asian studies.

Figures 2 and 3 summarize the findings of previous studies on the risk of CVD, CHD, heart failure, and stroke after PE and GH, respectively. Thirty-four studies were identified, the majority from Europe (18 studies^7–21^) and North America (nine studies^22–30^). Five studies were based in Asia^31–35^ and two in Australia.^36,37^ No studies based in South America or Africa were found. Only one of the identified studies was from a LMIC.^33^

The majority of studies were among white populations or populations with mixed ethnicities, but results were not stratified by ethnicity. Therefore, the comparison of the presence and magnitude of cardiovascular risk across different ethnicities was limited.

Figure 2 summarizes risk of CVD outcomes after PE. A higher CVD risk after PE was reported in most European and all North American studies, which was consistent across ethnicities. The risk was highest in a population in North America: RR=2.55 (1.70–3.83).^25^ The presence of CHD risk after PE was not as consistent across different continents. Most European populations were found to have a higher CHD risk after PE, as did one Australian and one Asian population. The highest reported risk was reported in an Iranian population: RR = 7.17 (3.09–16.63).^33^ However, in one North American population, the risk of CHD was not higher for women with PE than women without PE, and the higher CHD risk observed in two other North American populations was not significant. A higher risk of heart failure after PE was found in four out of six European populations and one Asian population, with the highest risk reported in a European population: RR = 8.84 (1.22–64.02).^38^ The two other European populations had non-significantly higher risk of heart failure after PE. An increased stroke risk after PE was found in two of the five European populations. A third European population was also observed to have a higher stroke risk although this was not significant, and two European populations had little evidence of an increased stroke risk after PE. One Australian population was not found to have a significantly higher risk, but in both Asian populations a higher stroke risk was found.

Figure 3 summarizes risk of CVD outcomes after GH. CVD risk was higher after GH for all except three European populations, and three out of four North American populations, with the highest risk reported in a European population, RR=12.77 (6.78–24.04).^39^ No difference in CVD risk between women with prior GH and women without GH was found in two European and one North American population. CHD risk after GH was higher in most European populations, one North American population, and one Australian population. The highest risk was reported in a European population: RR = 4.00 (2.21–7.24).^11^ There was inconsistent evidence for a higher risk of heart failure after GH. Two European populations were found to have a higher risk, while no significantly higher risk of heart failure was observed in two European populations and one North American population. A higher stroke risk after GH was found in four out of five European populations and one Asian population, with the highest reported risk in a European population: RR = 7.99 (2.45–26.07). However, in an Australian population, no higher risk of stroke after GH was observed.

In general, there was broadly consistent evidence that PE is associated with a higher risk of CVD, CHD, heart failure, and stroke, in the majority of populations studied. GH was also associated with a higher risk of CVD, CHD, and stroke in most populations although the evidence for a higher risk of heart failure was not consistently found.

### Shared Mechanisms or Cause and Effect?

Pregnancy is purported to be a “stress test”^40^ for a woman’s body as the additional hemodynamic, metabolic, and hormonal demands may unmask a predisposition to developing CVD later in life. Despite the negative connotations suggesting development of GH or PE is “failure,” it is a commonly used analogy for understanding the links between HDP and CVD.

Evidence in support of this theory is prevalent with many CVD risk factors shared with GH or PE (Supplementary Figure 1) although cardiovascular risk factors may not fully explain the risk of CVD after PE.^41^

PE and GH may also damage the cardiovascular system, thus contributing to CVD development. Inflammatory stress, coagulation dysregulation, and endothelial damage arising from complicated pregnancies may result in vascular damage.^42^ The presence of a dose-response relationship between PE and CVD^43^ supports this theory, as additional pregnancies affected by PE would cause greater damage and therefore increase CVD risk decades after pregnancy.

Furthermore, the timing of diagnosis of these conditions during pregnancy is important. Drawing from the aforementioned theories, a diagnosis earlier in pregnancy, when physiological demands are lower, suggests the underlying conditions are at a more adverse stage, or that greater damage has occurred. Therefore, an earlier diagnosis of GH or PE may be associated with a greater subsequent CVD risk. Previous work supports this theory, with greater changes in angiogenic factors in women with early-onset compared to late-onset PE.^44^

### Interventions

Having interventions to mitigate CVD risk after GH or PE is imperative but few trials have been conducted. One previous review of this topic covering studies from 2008–2019 identified only two trials.^45^ A trial of calcium vs. placebo in 201 postpartum women with prior PE reported a non-significant trend toward decreased blood pressure. The second randomized control trial (RCT) enrolled 151 women with PE in the previous 5 years into either an online education program about CVD risk factors and personalized phone-based lifestyle coaching or general information for the control group. The intervention group had increased knowledge of CVD risk factors, greater healthy eating, and decreased physical inactivity.

After this review, two trials have published results. A pilot trial, Be Healthe for Your Heart, sent weekly emails to support improvements in CVD risk factors using behavior change techniques (e.g. self-monitoring and goal setting) to women with a recent history of PE. It did not report improved CVD risk, CVD risk factors, or health behaviors after 3 months.^46^ Heart Health 4 New Moms, a 9-month behavioral intervention trial of women with prior PE included online educational modules, a community forum, heart-healthy resources, and communication with a lifestyle coach. It found intervention participants reported increased healthy eating, greater knowledge of CVD risk factors, and less physical inactivity than participants in the control arm.^47^ Additional trials that may provide further insight on blood pressure management strategies, exercise, aspirin, lifestyle intervention programs, and statins are currently underway.^48–50^

Prenatal interventions that may moderate vascular damage have been assessed in HIC. These include managing adverse levels of cardiovascular risk factors in pregnancy, such as lifestyle changes that limit excess gestational weight gain, a risk factor for PE and GH.^51^ However, although these interventions are effective,^51^ benefits may be modest.^52^

Postpartum interventions are also an option. The World Health Organization and national bodies, including the American College of Obstetricians and Gynecologists (ACOG) and the National Institute for Health and Clinical Excellence (NICE), recommend that all women be offered postpartum follow-up care up to 12 weeks after delivery, providing a potential time point for cardiovascular intervention or for referral from obstetrics to primary care or cardiology.^53–55^ The extent of postpartum and ongoing care varies between countries and is likely to be impacted by the duration of paid parental leave available to mothers. In HIC, this varies between 6.5 weeks (United Arab Emirates) to 68.5 weeks (Sweden). A longer parental leave will facilitate access to healthcare providers.

Regardless of the duration or availability of care after birth, routine referral from obstetrics to cardiology is not in place in many countries, and not all women attend antenatal appointments when offered, with the proportion of women with and without prior pregnancy complications attending postpartum care as low as 52% in some settings.^56^

### Expanding Intervention Opportunities in High-Income Countries

The majority of national and international bodies that have provided guidance on cardiovascular follow-up after a hypertensive disorder of pregnancy are from HIC (Supplementary Table 1). However, most international healthcare systems treat pregnancy as an isolated life event, and obstetric information is not readily available to different healthcare disciplines. In HIC, calls for action have been made to disrupt these healthcare silos and create multidisciplinary teams that deliver comprehensive care by bringing together professionals from different disciplines to work together.^57^

This multidisciplinary approach remains theoretical for the most part. Opportunities to intervene before CVD problems arise are missed, as are other benefits, including reduced healthcare utilization and costs. However, coordination between healthcare providers is feasible. For example, cardio-obstetric care could be integrated by including CVD risk factor assessment for women when children attend well-child visits or receive immunizations.^58^

If women are referred to primary care or cardiology, it is important to consider what constitutes quality follow-up care. Counseling strategies implemented in the postpartum period could include discussion of heart age calculations.^59^ Interventions have been proposed to improve the cardiovascular risk factor profile of postpartum women, including lifestyle education programs and peer support.^45^ However, interventions are not always as effective as desired. A review of mostly Western European and North American studies found behavioral interventions that promoted self-regulation decreased energy intake, but this did not consistently translate to improvements in weight loss and participation in physical activity.^60^

### Expanding Intervention Opportunities in Low- and Middle-Income Countries

Opportunities for cardiovascular intervention in LMIC differ from those in HIC. Integral steps are needed to address the long-term needs of women who have experienced HDP or other pregnancy complications. Of most pressing concern is the early identification and diagnosis of HDP and other obstetric conditions, such as gestational diabetes. Over-burdened health systems and out-of-pocket costs limit the potential for scheduled hospital visits. Therefore, community healthcare workers may be a critical player for addressing this need. It is important to build the capacity of community healthcare workers to identify and manage women with PE and GH and thus limit the need to refer women to hospitals for management and identification of PE and GH.

Further steps that are integral to addressing the long-term cardiovascular and broader health needs of women in LMIC include (1) electronic health records systems to facilitate data exchange between different sectors of the healthcare system; (2) healthcare approaches that incorporate broad health determinants including housing, waste management, and education, which will have benefits beyond CVD; (3) research funding that encourages long-term CVD interventions in LMIC, which should be determined by the needs of individual countries; and (4) long-term support for frugal healthcare innovation (effective solutions to common problems with minimal use of resources) to allow societies to promote health while minimizing out-of-pocket costs.

Frugal innovations may include utilizing existing activities that impact child survival and health to also reduce CVD risk. For example, micronutrient supplementation in early childhood could be expanded to include the mother, as poor nutrition is linked to CVD.^61^ Further promotion of breastfeeding could improve infant outcomes and reduce the risk of CVD risk factors, such as Type 2 diabetes and hypertension.^62^

### Future Directions for Research

In HIC, additional research is needed in non-white populations to assess the epidemiology. It is also important to identify which interventions reduce CVD risk for women with previous GH or PE.^63^ Improving communication between patients and healthcare providers is likely to be an essential component of these approaches.

Identification and implementation of interventions is only appropriate once the epidemiology of CVD risk after GH or PE is understood. This is not well understood in LMIC despite these countries being disproportionately affected by GH, PE, and CVD.^3^ Only one study from a LMIC (Iran) was identified in this review.^33^ There is a clear rationale and need for investigating differences in the CVD risk after GH or PE in LMIC.

## Conclusions

PE and GH provide valuable information regarding how effectively a woman’s cardiovascular system adapts to the physiological demands of pregnancy, which has implications for CVD risk assessment. The period around childbearing offers a unique time in a woman’s life to understand and change the course of her chronic disease trajectory. In HIC, women with GH or PE should be aware of their higher CVD risk, but further research is needed to identify effective strategies to reduce their risk. In LMIC, there is a paucity of research on CVD risks after GH or PE. Thus, more studies are needed to understand the epidemiology of the association in these populations and frugal innovation will be needed to reduce CVD risk in populations with fragile healthcare systems.

## Supplemental Material

sj-pdf-1-ajl-10.1177_15598276211037964 – Supplemental Material for Maternal Cardiovascular Disease After Pre-Eclampsia and Gestational Hypertension: A Narrative Review Click here for additional data file. Supplemental Material, sj-pdf-1-ajl-10.1177_15598276211037964 for Maternal Cardiovascular Disease After Pre-Eclampsia and Gestational Hypertension: A Narrative Review by Clare Oliver-Williams, Jasmine D. Johnson and Catherine J. Vladutiu in American Journal of Lifestyle Medicine
