# Supplementary material for: Maternal Cardiovascular Disease After Pre-Eclampsia and Gestational Hypertension: A Narrative Review
Source: Am J Lifestyle Med. 2021 Dec 27;17(1):8–17. doi: 10.1177/15598276211037964 (PMC9830232; doi:10.1177/15598276211037964)
Supplement: sj-pdf-1-ajl-10.1177_15598276211037964 – Supplemental Material for Maternal Cardiovascular Disease After Pre-Eclampsia and Gestational Hypertension: A Narrative Review [file sj-pdf-1-ajl-10.1177_15598276211037964.pdf]

Online Supplement

eTable 1: Guidance on cardiovascular follow-up after a hypertensive disorder of pregnancy in national and international guidelines

Guidelines	Country	Follow-up CVD risk
**WHO, 2011**[1]	International	Not included.
**International Society for the study of Hypertension in Pregnancy (ISSHP)**[2]	International	All women with GH or PE require lifelong follow-up because of increased cardiovascular risk. Advise women with a GH or PE of their increased risks compared with women who have had normotensive pregnancies
**American College of Obstetricians and Gynecologists (ACOG), 2018**[3]	USA	Women with recurrent PE: annual blood pressure, lipids, fasting glucose and BMI measured. No recommendations on starting time or clinical assessment lead
**American Stroke Association, 2014**[4]	USA	Consider evaluating and treating all women with prior PE for cardiovascular risk factors (hypertension, obesity, smoking and dyslipidemia), starting 0.5-1 year post-partum.
**American Heart Association (AHA), 2011**[5]	USA	Appropriate referral postpartum by the obstetrician to a primary care physician or cardiologist so that in the years after

		pregnancy, risk factors can be carefully monitored and controlled. Healthcare professionals who meet women later in their lives should take a detailed history of pregnancy complications.
**Canadian Hypertensive Disorders of Pregnancy Working Group, 2014**[6]	Canada	Counsel women with previous HDP about the greater CVD risk, and adopting a healthy diet and lifestyle. Assessing CVD risk factors may be beneficial at 6 weeks post-partum.
**National Institute for Health and Care Excellence (NICE), 2017**[7]	UK	Advise women with a prior HDP of their increased risk of CVD in later life. Discuss lifestyle changes to reduce their risk with their GP or specialist.
**European Society of Cardiology/European Society of Hypertension (ESC/ESH), 2018**[8]	International	Annual check of blood pressure and metabolic factors by primary care physician for women with a HDP
**Society of Obstetric Medicine Australia and New Zealand (SOMANZ), 2014**[9]	Australia and New Zealand	Cardiovascular risk assessment every 5 years. Counselling women with prior PE that they will benefit from avoiding smoking, maintaining a healthy weight, exercising regularly and eating a healthily. Annual blood pressure check and regular (5 yearly or more

		frequent if indicated) assessment of other cardiovascular risk factors including serum lipids and blood glucose is recommended.
**Institute of Obstetricians and Gynaecologists, 2016**[10]	Ireland	Women with HDP should be counselled about their CVD risk and to minimize risk factors. Annual blood pressure checks and regular assessment of other cardiovascular risk factors (lipids, blood glucose) is recommended.
**The French Society of Hypertension, an affiliate of the French Society of Cardiology**[11]	France	Patients with HDP should be referred to a consultant post-partum to discuss CVD risk and how to manage CVD risk factors through a multidisciplinary care-plan.

BMI – body mass index; CVD – Cardiovascular Disease; GH – Gestational Hypertension; GP – general practitioner; HDP – hypertensive disorder of pregnancy; PE – pre-eclampsia.

eTable 2: Characteristics of studies assessing risk of Cardiovascular Disease, Coronary Heart Disease, Heart Failure and Stroke after pre-eclampsia, by country

Author, year of publication	Study Design	Population source & Ethnicity	No. of participants	Follow-up duration, years	Mean age at baseline, years	Results	Covariates controlled for
Cardiovascular Disease							
Arnaout 2018	Cohort	North America - Mixed: non-Hispanic white: 35%, African–American: 6%, Hispanic: 39%, Asian or Pacific Islanders: 12%	1662045	2.7	28	HR=2.39 (95%:1.97-2.89)	age, race, insurance status, median household income, chronic kidney disease, pre-existing diabetes, obesity, drug abuse, smoking, multiple gestations.
Bhattacharya 2012	Cohort	Europe – White	34854	34.5	24	IRR=1.30 (95%CI:1.06-16.0)	Year of birth, social class and smoking
Cain 2016	Cohort	North America - Mixed Non-Hispanic white 49%; Non-Hispanic black 18%; Hispanic 27%; Non-Hispanic other 5%	302686	4.9	25.1	HR=1.42 (95%CI:1.14-1.76)	Age, race/ethnicity, nativity, education, income, 5-year history of hyperlipidemia, migraine, lupus; pre-pregnancy BMI, gestational diabetes, tobacco use, drug use, and infant sex
Cirillo 2015	Cohort	North America - African American	10721	Range: 44-52	26	HR=1.7 (95%CI:1.1-2.7)	Age, race, parity, BMI, and cigarette smoking
Cirillo 2015	Cohort	North America - Mixed – 67% Caucasian, 23% African American, 3% Hispanic, 4% Asian, 3% Other	10721	Range: 44-52	26	HR=2.20 (95%CI:1.34-3.60)	Age, race, parity, BMI, and cigarette smoking
Grandi 2017	Cohort	Europe - Unknown	146000	4.7	29.24	HR=0.6 (95%CI:0.2-1.8)	Age, smoking, BMI, alcohol use, year of cohort entry, region of residence, multiple gestation at 1st

							pregnancy, depression, dyslipidaemia, venous thromboembolism, polycystic ovary syndrome, gestational diabetes, diabetes mellitus, renal disease, migraines, family history of hypertension and cardiovascular disease, number of distinct drug classes prescribed, and use of statin, aspirin and anti-depressant medications
Haug 2019	Cohort	Europe - White	23885	18	24	HR=1.74 (95CI:1.00-3.02)	Age, highest obtained educational level, ever smoked daily, parity at age 40, maternal birth year, family history of coronary heart disease
Kestenbaum 2003	Nested case-control	North America - Mixed – White: 80%, African American: 3%, Hispanic: 9%, Other: 8%	103589	7.8	26.2	OR=2.55 (95%CI:1.70-3.83)	Age, parity, calendar year of delivery
Lykke 2010	Cohort	Europe – White	782287	14.8	26.8	HR=2.16 (95%CI:1.79-2.61)	Maternal age & year of delivery
Markovitz 2019	Cohort	Europe – White	18231	8.2	52	HR=1.60 (95%CI:1.16-2.17)	Age, Age^2, Systolic blood pressure, Serum total cholesterol, daily smoking, antihypertensives, low HDL cholesterol, family history of premature MI, gestational hypertension, preterm birth, small for gestational age.
Männistö 2013	Cohort	Europe - White	12055	39.4	26.6	HR=1.40 (95%CI:1.11-1.76)	Age, pre-pregnancy BMI, pre-pregnancy smoking, parity, and diabetes mellitus and socioeconomic status.
Ray 2005	Cohort	North America - Unknown	963263	8.7	28	HR=2.1 (95%CI:1.8-2.4)	Age, multiple gestation, length of stay, income quintile, rural residence, drug dependence, and gestational diabetes mellitus in index delivery, and hypertension, any diabetes mellitus, obesity, dyslipidemia, tobacco use, renal disease, migraine headache, and systemic lupus erythematosus
Riise 2019	Cohort	Europe – Unknown	20075	11.4	26	IRR=2.2 (95%CI:1.7-2.7)	Age at recruitment age at first delivery, education (primary, high school/vocational, any college/ university) and a family history of MI prior to age 60
Schmiegelow 2014	Cohort	Europe - Mixed – majority White	273101	4.5	30.4	HR=2.56 (95%CI:1.67-	Age, smoking, and year of inclusion

						3.93)	
Skjaerven 2012	Cohort	Europe – White	836147	25	Not given	HR=1.9 (95% 1.6-2.2)	Age at first birth, year of first birth, education.
Coronary Heart Disease							
Andolf 2017	Cohort	Europe - 93.6% White	284598	35	26.1	HR=1.50 (95%CI:1.11-2.04)	age at birth, educational level in 1985, marital status and origin (Nordic/non-Nordic), history of cardiovascular disease later in life (diabetes, arteriosclerosis, stroke, ischemic heart disease, heart failure and hypertension).
Bhattacharya 2012	Cohort	Europe – White	34854	34.5	24	IRR=1.38 (95CI:1.03-1.84)	Year of birth, social class and smoking
Borna 2011	Case-control	Asia - Iranian	690	Not applicable	57	OR=7.17 (95%CI:3.09-16.62)	None
Haukkamaa 2009	Cross-sectional	Europe – Unknown	767	Not applicable	57	OR=3.63 (95%CI:1.50-8.79)	None
Lykke 2009	Cohort	Europe – White	782287	14.6	26.8	HR=1.58 (95CI:1.46-1.71)	Maternal age, year of delivery, preterm delivery, small for gestational age offspring, placental abruption, stillbirth, and type 2 diabetes mellitus
M[c]Donald 2013	Cohort	North America - White	327	20	63	RR=0.91 (95%CI:0.54-1.52)	Age, albuminuria, education, ethnicity, GFR<60, HRT, Interheart risk score
Männistö 2013	Cohort	Europe – White	12055	39.4	26.6	HR=1.36 (95CI:1.01-1.83)	Age, pre-pregnancy BMI, pre-pregnancy smoking, parity, and diabetes mellitus and socioeconomic status.
Ray 2015	Cohort	North America - Unknown	1985	5	32	HR=1.61 (95%CI:1.00-2.58)	Age, income quintile, rural residence, parity, type of index coronary artery revascularization (PCI or CABG), time between the last obstetrical delivery and index coronary artery revascularization and chronic hypertension, diabetes, obesity, dyslipidemia, tobacco or drug dependence or kidney disease
Riise 2019	Cohort	Europe – Unknown	20075	11.4	26	IRR=1.8 (95%CI:0.9-3.4)	Age at recruitment age at first delivery, education (primary, high school/vocational, any college/ university) and a family history of MI prior to age 60
Sia 2019	Case-	North America -	490	Not	66.3	OR=1.86	BMI, smoking status, diabetes, dyslipidemia,

	control	White		applicable		(95%CI:0.97-3.57)	hypertension, parity, family history of CVD, prior preterm birth, abortions and miscarriages, prior gestational hypertension and small for gestational age.
Skjaerven 2012	Cohort	Europe – White	836147	25	Not given	HR=2.09 (95%:1.56-2.82)	Age at first birth, year of first birth, education.
Tooher 2017	Cohort	Australia - Unknown	27887	20	27	OR=2.67 (95%CI:1.49-4.81)	Age, gestation, and parity
Wikstrom 2005	Cohort	Europe – Unknown	391017	Max: 15	Range: 15-64	IRR=2.8 (95%CI:2.2-3.7)	Age, socio-economic level and category of hospital
Wilson 2003	Cohort	Europe – White	3593	Not given	24.2	RR=0.89 (95%CI:0.56-1.40)	age at delivery and social class.
Heart Failure							
Andolf 2017	Cohort	Europe - 93.6% White	284598	35	26.1	HR=1.28 (95%CI:0.71-2.31)	age at birth, educational level in 1985, marital status and origin (Nordic/non-Nordic), history of cardiovascular disease later in life (diabetes, arteriosclerosis, stroke, ischemic heart disease, heart failure and hypertension).
Arnaout 2018	Cohort	North America - Mixed: non-Hispanic white: 35%, African–American: 6%, Hispanic: 39%, Asian or Pacific Islanders: 12%	1662045	2.7	28	HR=3.0 (95%CI: 2.7-3.4)	age, race, insurance status, median household income, chronic kidney disease, pre-existing diabetes, obesity, drug abuse, smoking, multiple gestations.
Breetveld 2017	Case-control	Europe - Mixed – most European	103	Not applicable	36	OR=8.84 (95CI:1.22-64.00)	None
Haug 2019	Cohort	Europe – White	23885	18	24	HR=1.74 (95CI:1.00-3.02)	Age, highest obtained educational level, ever smoked daily, parity at age 40, maternal birth year, family history of coronary heart disease
Kuo 2018	Cohort	Asia - Taiwanese	6475	9.8	29.7	HR=7.39 (95%CI:2.86-19.06)	Age, diabetes, dyslipidemia
Lykke 2009	Cohort	Europe – White	782287	14.6	26.8	HR=1.68	Maternal age, year of delivery, preterm delivery,

						(95CI:1.44-1.95)	small for gestational age offspring, placental abruption, stillbirth, and type 2 diabetes mellitus
Männistö 2013	Cohort	Europe – White	12055	39.4	26.6	HR=1.69 (95CI:1.12-2.56)	Age, pre-pregnancy BMI, pre-pregnancy smoking, parity, and diabetes mellitus and socioeconomic status.
Melchiorre 2011	Case-control	Europe - Mixed – 66.4% White	77	Not applicable	33.5	OR=4.27 (95CI:2.09-8.70)	None
Stroke							
Andolf 2017	Cohort	Europe - 93.6% White	284598	35	26.1	HR=0.91 (95%CI:0.56-1.46)	age at birth, educational level in 1985, marital status and origin (Nordic/non-Nordic), history of cardiovascular disease later in life (diabetes, arteriosclerosis, stroke, ischemic heart disease, heart failure and hypertension).
Arnaout 2018	Cohort	North America - Mixed: non-Hispanic white: 35%, African–American: 6%, Hispanic: 39%, Asian or Pacific Islanders: 12%	1662045	2.7	28	HR=2.3 (95%CI:1.8-3.0)	age, race, insurance status, median household income, chronic kidney disease, pre-existing diabetes, obesity, drug abuse, smoking, multiple gestations.
Kuo 2018	Cohort	Asia - Taiwanese	6475	9.8	29.7	HR=3.47 (95%CI:1.46-8.23)	Age, diabetes, dyslipidemia
Lin 2016	Cohort	Asia – Taiwanese	36950	Max: 13	31.06	IRR=2.19 (95%CI:1.22-3.90)	Age
Lykke 2009	Cohort	Europe - White	782287	14.6	26.8	HR=1.45 (95%CI: 1.32-1.59)	Maternal age, year of delivery, preterm delivery, small for gestational age offspring, placental abruption, stillbirth, and type 2 diabetes mellitus
Männistö 2013	Cohort	Europe – White	12055	39.4	26.6	HR=1.19 (95CI:0.68-2.09)	Age, pre-pregnancy BMI, pre-pregnancy smoking, parity, and diabetes mellitus and socioeconomic status.
Schmiegelow 2014	Cohort	Europe - Mixed – majority White	273101	4.5	30.4	HR=5.18 (95%CI:2.03-13.22)	Age, smoking, and year of inclusion
Tooher 2017	Cohort	Australia – Unknown	27887	20	27	OR=2.03 (95%CI:0.75-	Age, gestation, and parity

						5.49)	
Wilson 2003	Cohort	Europe – White	3593	Not given	24.2	RR=3.41 (95%CI:0.95-12.20)	age at delivery and social class.

IRR – incidence rate ratio; HR – hazard ratio; OR – odds ratio; RR – relative risk

eTable 3: Characteristics of studies assessing risk of Cardiovascular Disease, Coronary Heart Disease, Heart Failure and Stroke after gestational hypertension, by country

Author, year of publication	Study type	Population source & Ethnicity	No. of participants	Follow-up duration, if applicable	Mean age at baseline	Results	Covariates controlled for
Cardiovascular Disease							
Arnaout 2018	Cohort	North America - Mixed: non-Hispanic white: 35%, African–American: 6%, Hispanic: 39%, Asian or Pacific Islanders: 12%	1662045	2.7	28	HR=1.76 (95%CI:1.36-2.27)	age, race, insurance status, median household income, chronic kidney disease, pre-existing diabetes, obesity, drug abuse, smoking, multiple gestations.
Bhattacharya 2012	Cohort	Europe – White	34854	34.5	24	IRR=0.90 (95%CI:0.62-1.30)	Year of birth, social class and smoking
Cain 2016	Cohort	North America - Mixed Non-Hispanic white 49%; Non-Hispanic black 18%; Hispanic 27%; Non-Hispanic other 5%	302686	4.9	25.1	HR=0.99 (95%CI:0.85-1.16)	Age, race/ethnicity, nativity, education, income, 5-year history of hyperlipidemia, migraine, lupus; pre-pregnancy BMI, gestational diabetes, tobacco use, drug use, and infant sex
Grandi 2017	Cohort	Europe - Unknown	146000	4.7	29.24	HR=2.3 (95%CI:1.8-2.9)	Age, smoking, BMI, alcohol use, year of cohort entry, region of residence, multiple gestation at 1st pregnancy, depression, dyslipidaemia, venous thromboembolism, polycystic ovary syndrome, gestational diabetes, diabetes mellitus, renal disease, migraines, family history of hypertension and cardiovascular disease, number of distinct drug classes prescribed, and use of statin, aspirin and anti-depressant

							medications
Grandi 2018	Cohort	Europe - Unknown	146748	4.7	29.24	HR=2.12 (95%CI:1.66-2.70)	Age, smoking, obesity, excessive alcohol use, year of cohort entry, region of residence, multifetal gestation at first pregnancy, depression, dyslipidemia, venous thromboembolism, polycystic ovary syndrome, gestational diabetes, diabetes mellitus, renal disease, migraines, family history of hypertension and family history of CVD, number of distinct drug classes prescribed and use of statins, aspirin and anti-depressants
Haug 2019	Cohort	Europe – White	23885	18	24	HR=1.30 (95%CI:1.00-1.68)	Age, highest obtained educational level, ever smoked daily, parity at age 40, maternal birth year, family history of coronary heart disease
Kestenbaum 2003	Nested case-control	North America - Mixed – White: 80%, African American: 3%, Hispanic: 9%, Other: 8%	103589	7.8	26.2	OR=2.8 (95%CI:1.6-4.8)	Age, parity, calendar year of delivery
Luoto 2008	Cohort	Europe – White	4000	44	Not given	HR=0.90 (95%CI:0.62-1.30)	Age, hormone use, height, marital status and visit to private doctor
Lykke 2010	Cohort	Europe – White	782287	14.8	26.8	HR=2.47 (95%CI:1.74-3.52)	Maternal age & year of delivery
Männistö 2013	Cohort	Europe – White	12055	39.4	26.6	HR=0.73 (95CI:0.46-1.15)	Age, pre-pregnancy BMI, pre-pregnancy smoking, parity, and diabetes mellitus and socioeconomic status.
Markovitz 2019	Cohort	Europe – White	18231	8.2	52	HR=1.25 (95%CI:1.11-1.41)	Age, Age^2, Systolic blood pressure, Serum total cholesterol, daily smoking, antihypertensives, low HDL cholesterol, family history of premature MI, pre-eclampsia,

							preterm birth, small for gestational age.
Ray 2005	Cohort	North America - Unknown	963263	8.7	28	HR=1.8 (95%CI:1.4-2.2)	Age, multiple gestation, length of stay, income quintile, rural residence, drug dependence, and gestational diabetes mellitus in index delivery, and hypertension, any diabetes mellitus, obesity, dyslipidemia, tobacco use, renal disease, migraine headache, and systemic lupus erythematosus
Riise 2018	Cohort	Europe – Unknown	617589	14.3	26.3	HR=1.8 (95%CI:1.7-2.0)	Age, educational level, marital status, and birth year of first child
Riise 2019	Cohort	Europe – Unknown	20075	11.4	26	IRR=2.5 (95%CI:1.8-3.4)	Age at recruitment age at first delivery, education (primary, high school/vocational, any college/ university) and a family history of MI prior to age 60
Schmiegelow 2014	Cohort	Europe - Mixed – majority White	273101	4.5	30.4	HR=12.77 (95%CI:1.47-5.21)	Age, smoking, and year of inclusion
Yeh 2014	Nested case-control	Asia - Taiwanese	5765	5.8	29.8	HR=2.44 (95%CI:1.80-3.31)	Age, diabetes, dyslipidaemia, incident hypertension, date of delivery
Coronary Heart Disease							
Andolf 2017	Cohort	Europe - 93.6% White	284598	35	26.1	HR=1.26 (95%CI:1.13-1.40)	age at birth, educational level in 1985, marital status and origin (Nordic/non-Nordic), history of cardiovascular disease later in life (diabetes, arteriosclerosis, stroke, ischemic heart disease, heart failure and hypertension).
Bhattacharya 2012	Cohort	Europe – White	34854	34.5	24	IRR=1.35 (95CI:1.15-1.59)	Year of birth, social class and smoking
Borna 2011	Case-control	Asia - Iranian	690	Not applicable	57	OR=3.05 (95%CI:1.88-4.94)	None

Haukkamaa 2009	Cross-sectional	Europe - Unknown	767	Not applicable	57	OR=4.0 (95%CI:2.21-7.25)	None
Lykke 2009	Cohort	Europe – White	782287	14.6	26.8	HR=1.48 (95%CI:1.25-1.76)	Maternal age, year of delivery, preterm delivery, small for gestational age offspring, placental abruption, stillbirth, and type 2 diabetes mellitus
Männistö 2013	Cohort	Europe – White	12055	39.4	26.6	HR=1.44 (95CI:1.24-1.68)	Age, pre-pregnancy BMI, pre-pregnancy smoking, parity, and diabetes mellitus and socioeconomic status.
Riise 2018	Cohort	Europe - Unknown	617589	14.3	26.3	HR=1.7 (95%CI:1.3-2.1)	Age, educational level, marital status, and birth year of first child
Riise 2019	Cohort	Europe – Unknown	20075	11.4	26	IRR=2.2 (95%CI:1.0-5.1)	Age at recruitment age at first delivery, education (primary, high school/vocational, any college/ university) and a family history of MI prior to age 60
Sia 2019	Case-control	North America – White	490	Not applicable	66.3	OR=3.34 (95%CI:1.03-10.9)	BMI, smoking status, diabetes, dyslipidemia, hypertension, parity, family history of CVD, prior preterm birth, abortions and miscarriages, prior preeclampsia and small for gestational age.
Tooher 2017	Cohort	Australia – Unknown	27887	20	27	OR=3.19 (95%CI:2.11-4.83)	Age, gestation, and parity
Wilson 2003	Cohort	Europe – White	3593	Not given	24.2	RR=1.06 (95%CI:0.68-1.65)	age at delivery and social class.
Wikstrom 2005	Cohort	Europe - Unknown	391017	Max: 15	Range: 15-64	IRR=1.6 (95%CI:1.3-2.1)	Age, socio-economic level and category of hospital
Heart Failure							
Andolf 2017	Cohort	Europe - 93.6% White	284598	35	26.1	HR=1.25 (95%CI:1.11-	age at birth, educational level in 1985, marital status and origin

						1.41)	(Nordic/non-Nordic), history of cardiovascular disease later in life (diabetes, arteriosclerosis, stroke, ischemic heart disease, heart failure and hypertension).
Arnaout 2018	Cohort	North America - Mixed: non-Hispanic white: 35%, African–American: 6%, Hispanic: 39%, Asian or Pacific Islanders: 12%	1662045	2.7	28	HR=1.4 (95%CI: 1.0-2.0)	age, race, insurance status, median household income, chronic kidney disease, pre-existing diabetes, obesity, drug abuse, smoking, multiple gestations.
Haug 2019	Cohort	Europe – White	23885	18	24	HR=0.96 (95%CI:0.42-2.18)	Age, highest obtained educational level, ever smoked daily, parity at age 40, maternal birth year, family history of coronary heart disease
Lykke 2009	Cohort	Europe – White	782287	14.6	26.8	HR=1.37 (95%CI:0.98-1.93)	Maternal age, year of delivery, preterm delivery, small for gestational age offspring, placental abruption, stillbirth, and type 2 diabetes mellitus
Männistö 2013	Cohort	Europe – White	12055	39.4	26.6	HR=1.78 (95CI:1.43-2.21)	Age, pre-pregnancy BMI, pre-pregnancy smoking, parity, and diabetes mellitus and socioeconomic status.
Stroke							
Andolf 2017	Cohort	Europe - 93.6% White	284598	35	26.1	HR=1.30 (95%CI:1.14-1.48)	age at birth, educational level in 1985, marital status and origin (Nordic/non-Nordic), history of cardiovascular disease later in life (diabetes, arteriosclerosis, stroke, ischemic heart disease, heart failure and hypertension).
Arnaout 2018	Cohort	North America - Mixed: non-Hispanic white: 35%, African–American: 6%,	1662045	2.7	28	HR=1.4 (95CI:1.0-2.0)	age, race, insurance status, median household income, chronic kidney disease, pre-existing diabetes, obesity, drug abuse, smoking, multiple gestations.

		Hispanic: 39%, Asian or Pacific Islanders: 12%					
Lin 2016	Cohort	Asia - Taiwanese	36950	Max: 13	31.06	IRR=3.72 (95%CI:3.63-3.81)	Age
Lykke 2009	Cohort	Europe – White	782287	14.6	26.8	HR=1.51 (95%CI:1.26-1.81)	Maternal age, year of delivery, preterm delivery, small for gestational age offspring, placental abruption, stillbirth, and type 2 diabetes mellitus
Männistö 2013	Cohort	Europe – White	12055	39.4	26.6	HR=1.59 (95CI:1.24-2.04)	Age, pre-pregnancy BMI, pre-pregnancy smoking, parity, and diabetes mellitus and socioeconomic status.
Schmiegelow 2014	Cohort	Europe - Mixed – majority White	273101	4.5	30.4	HR=7.99 (95%CI:2.45-26.09)	Age, smoking, and year of inclusion
Tooher 2017	Cohort	Australia – Unknown	27887	20	27	OR=0.57 (95%CI:0.14-2.31)	Age, gestation, and parity
Wilson 2003	Cohort	Europe – White	3593	Not given	24.2	RR=2.42 (95%CI:0.59-9.98)	age at delivery and social class.

IRR – incidence rate ratio; HR – hazard ratio; OR – odds ratio; RR – relative risk

eFigure 1: Presence and Direction of Risk Factors for Cardiovascular Disease, Pre-eclampsia and Gestational Hypertension l

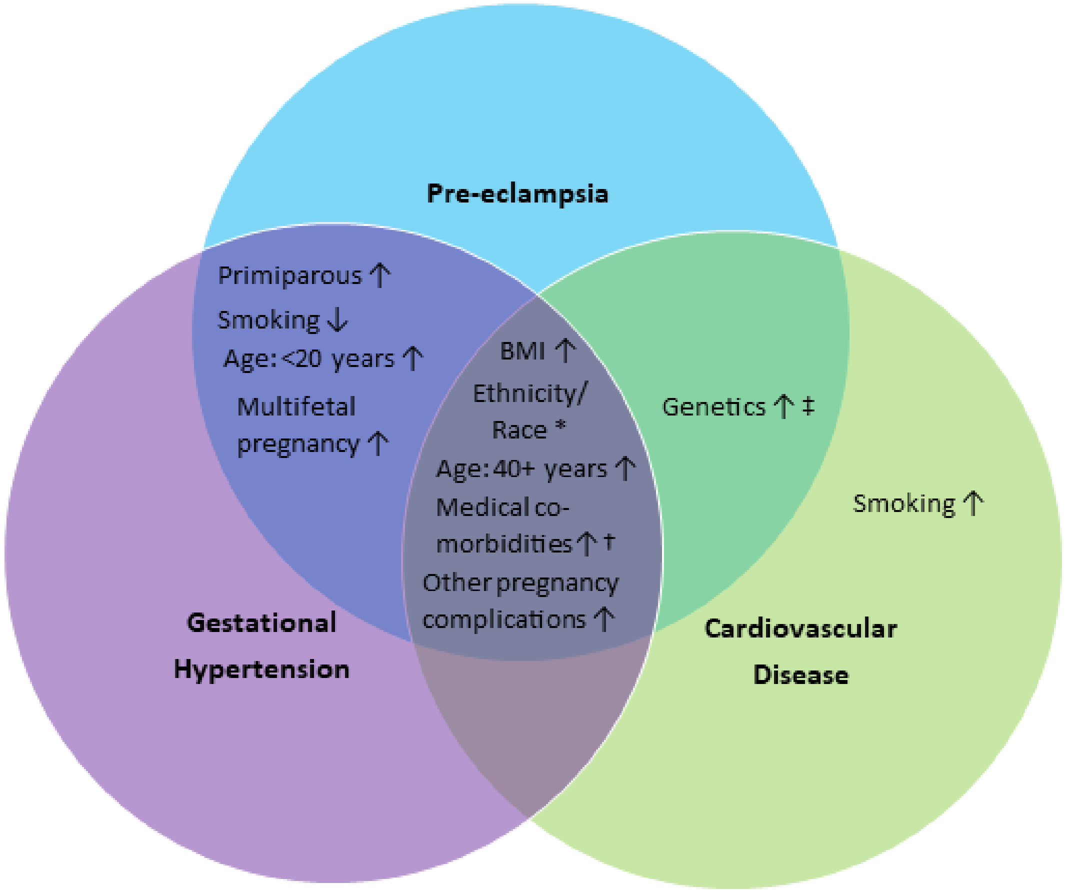

Details of Race/Ethnicity relationships (relative to White women): Chinese ↓; Māori; South Asian; Pacific Islander; Aboriginal Australian; African American ↑ The risk of HDP for Hispanic women is unclear.

† Co-morbidities include chronic hypertension; polycystic ovary syndrome; systemic lupus erythematosus; kidney injury and disease

‡ Certain genetic factors are known to increase the risk of PE and CVD, although they may not be shared by the two conditions

References

1 World Health Organization. Global atlas on cardiovascular disease prevention and control. Geneva, Switzerland: 2011. https://www.who.int/cardiovascular_diseases/publications/atlas_cvd/en/

2 Brown MA, Magee LA, Kenny LC, *et al.* The hypertensive disorders of pregnancy: ISSHP classification, diagnosis & management recommendations for international practice. Pregnancy Hypertens. 2018;**13**:291–310. doi:10.1016/j.preghy.2018.05.004

3 ACOG Practice Bulletin No. 202: Gestational Hypertension and Preeclampsia. *Obstet Gynecol* 2019;**133**. doi:10.1097/AOG.0000000000003018

4 Bushnell C, McCullough LD, Awad IA, *et al.* Guidelines for the prevention of stroke in women: a statement for healthcare professionals from the American Heart Association/American Stroke Association. *Stroke* 2014;**45**:1545–88. doi:10.1161/01.str.0000442009.06663.48

5 Mosca L, Benjamin EJ, Berra K, *et al.* Effectiveness-Based Guidelines for the Prevention of Cardiovascular Disease in Women—2011 Update. *Circulation* 2011;**123**:1243–62. doi:10.1161/CIR.0b013e31820faaf8

6 Magee LA, Pels A, Helewa M, *et al.* Diagnosis, evaluation, and management of the hypertensive disorders of pregnancy. Pregnancy Hypertens. 2014;**4**:105–45. doi:10.1016/j.preghy.2014.01.003

7 NICE. Recommendations for research | Hypertension in pregnancy: diagnosis and management | Guidance [NG133]. 2019. https://www.nice.org.uk/guidance/ng133

8 Regitz-Zagrosek V, Roos-Hesselink JW, Bauersachs J, *et al.* 2018 ESC Guidelines for the management of cardiovascular diseases during pregnancy. Eur. Heart J. 2018;**39**:3165–241. doi:10.1093/eurheartj/ehy340

9 Lowe SA, Bowyer L, Lust K, *et al.* SOMANZ guidelines for the management of hypertensive disorders of pregnancy 2014. *Aust N Z J Obstet Gynaecol* 2015;**55**:e1-29. doi:10.1111/ajo.12399

10 Royal College of Physicians of Ireland. Clinical Practice guideline No. 37. The management of hypertension in pregnancy. 2016. https://rcpi-live-cdn.s3.amazonaws.com/wp-content/uploads/2017/02/Hypertension-Guideline_approved_120716-1.pdf

11 Mounier-Vehier C, Amar J, Boivin JM, *et al.* Hypertension and pregnancy: expert consensus statement from the French Society of Hypertension, an affiliate of the French Society of Cardiology. *Fundam Clin Pharmacol* 2017;**31**. doi:10.1111/fcp.12254
